# Mania as Debut of Cushing's Syndrome

**DOI:** 10.1155/2020/9127632

**Published:** 2020-03-04

**Authors:** Ricardo Álvarez Martínez, Rosa María Tomé Rodríguez, María Álvarez Ariza, Carlos Spuch, Jose M. Olivares

**Affiliations:** ^1^Department of Psychiatry, Vigo Health Area, Hospital Álvaro Cunqueiro, Spain; ^2^Translational Neuroscience Group, Galicia Sur Health Research Institute, Spain; ^3^Centro de Investigación Biomédica en Red de Salud Mental (CIBERSAM), Spain

## Abstract

This is a case of a patient affected by Cushing syndrome that was admitted at the hospital due to hormonal problems. He had presented psychiatric symptoms that were mistakenly considered not directly connected to the pathology causing the clinical condition, but a mere psychological reaction to it.

## 1. Introduction

Cushing's syndrome is a hormonal disorder caused by excess glucocorticoids. In 70-80% of cases, this excess is due to an excess of ACTH, being the rest of them (10-20%) ACTH independent [1, 2]. The usual symptoms in these patients include central obesity, abdominal stretch marks, moon-shaped face, hirsutism, insulin resistance, and facial plethora, although clinical presentation may be variable [3]. Early diagnosis is very complex due to its low incidence and the lack of specificity of its clinical manifestations that can be seen in other pathologies, such as metabolic syndrome [4]. The elevation of hormones also influences brain functioning, and therefore, this syndrome can be accompanied by several psychiatric manifestations, being the most common irritability, anxiety, depression, and cognitive deterioration and to a lesser extent mania or hypomania and psychosis [1]. In some cases, psychiatric symptoms may even precede for months or years the onset of the classical physical symptoms [5]. Psychiatric manifestations may completely remit after the correct treatment of the underlying cause.

## 2. Case Presentation

A 37-year-old male was admitted to the Acute Psychiatry Unit for a manic condition in which sleep disorders (insomnia of conciliation and maintenance), dysphoria, inadequate attitude, and altered cognitions predominated. In the three months prior to admission, symptoms compatible with hypomania were described and treatment with lithium (800 mg) and olanzapine (10 mg) was initiated. For three years, the patient had also been in regular contact with the Department of Endocrinology, due to a mild hypertension and hypokalemia that received no treatment.

With progressive adjustments of the psychopharmacological treatment, a partial remission of the psychiatric symptomatology was achieved; olanzapine dose was maintained and risperidone (6 mg) and Rivotril (4 mg) were gradually introduced, reducing lithium treatment to 600 mg/day. High blood pressure levels persisted. An endocrinological study was then performed with specific tests for Cushing, and an elevated urinary cortisol (1719.94 micrograms/day) elevation of ACTH concentration (115 pg/ml) and altered dexamethasone suppression test (weak suppression of 20.66 *μ*g/100 ml and strong suppression of 4.19 *μ*g/ml) were detected, so the patient was transferred to the Endocrinology Department. NMR to check the pituitary did not show significant alterations, and the rest of the hormonal tests did not show pathological data including levels of metanephrine, normetanephrine, and 3-methoxytyramine with 257, 348, and 365 mg/day, respectively. He was, then, diagnosed of endogenous ACTH-dependent hypercortisolism of probable pituitary origin, and the psychiatric treatment was unchanged.

After being discharged, the patient gradually presented symptoms of anxiety and depression (apathy and clinophilia), partly modulated by his evident and progressive physical deterioration. The dose of risperidone was reduced to 3 mg in an attempt to achieve affective improvement without success. At the same time, follow-up was maintained at the Endocrinology Outpatient's Clinic; a catheterization of lower petrosal sinuses was performed without abnormal results, suggesting an ectopic secretion of ACTH. A thoracoabdominal CT was then performed, showing a segmental bilateral pulmonary thromboembolism without clinical repercussion and a 1.5 cm hypervascularity lesion that protruded in the posterior edge of the hepatic segment VI. This pointed to the differential diagnosis between haemangioma or possible hypervascularity tumour, not being able to make an anatomopathological diagnosis. After an urgent pneumology study, a diagnosis of venous thromboembolic disease was made; hepatic MRI was performed, suggesting the possibility of metastasis of a neuroendocrine tumour, because scintigraphy (administration of SPECT-CT with 99mTc-Tektrotyd, dose: 20 mCi) also showed a focal uptake at the height of the right adrenal gland, at the crossroads between liver tissue, vena cava, and apex of the gland. In the following weeks, the patient developed an important myopathy, a cushingoid phenotype with rounded face, and multiple falls and traumatisms (particularly on his left knee). One month and a half after hospital discharge, he is admitted again to the hospital.

During the second admission, prostration, extreme weakness, and severe protein malnutrition were the most relevant features. The patient was also suffering from pain in the left limb, so he was referred to a traumatologist who diagnosed a femoral head fracture. PET scans were performed which, except for hypermetabolism at the left hip level, did not show any focal lesions suggesting tumoural pathology. The hypothesis of a neuroendocrine tumour was maintained, and the hip fracture was surgically intervened. The endocrinologist started treatment for associated comorbidities: amlodipine for blood pressure correction, osteoporosis treatment, somatostatin analogues, and protein and protein supplements due to protein shortage. Again, the patient is evaluated by the psychiatry service to alleviate anxiety and sleep disorders, progressively decreasing the dose of neuroleptics (withdrawal of risperidone) and introducing antidepressant treatment with mirtazapine (15 mg/day).

During the evolution, a decrease in the concentration of ACTH (13.70 pg/ml) and in the concentration of cortisol (265.31 mg/day) can be observed, and there is no modification of the mass found at the liver, whose study is pending completion. Follow-up by imaging tests (liver MRIs with and without contrast) to date supports that the liver lesion is a benign lesion in relation to focal nodular hyperplasia (lesion of 18 mm). The pulmonary nodule continues to be studied with high-definition chest CT controls without having modified its characteristics.

## 3. Discussion

Hypercortisolism is a rare disease, affecting multiple systems, associated with high morbidity and mortality if not treated on time. The prognosis of the disease is affected mainly by difficulties in diagnosis and treatment, which remain a challenge today. Another problem associated with hyperactivity of the hypothalamus-pituitary adrenal axis is the presentation of psychiatric diseases, such as mood disorders, usually depression. However, manic symptoms are not common at the onset of Cushing's syndrome.

This clinical case reflects the importance of an adequate organic screening in the presence of psychopathological symptoms in a patient with an atypical picture and no previous remarkable antecedents, concomitant with high arterial pressure that had been present for years and ionic alterations (hypokalemia) of recent instauration that could indicate an organic origin symptom.

Psychiatric symptoms that appear in the context of a neuroendocrine disease often occur at an advanced stage of the disease. However, they can occur in very early stages and, as in the case we have presented, can even mean the debut of the disease. The signs and symptoms typical of Cushing's syndrome (central obesity, facial plethora, stress fractures, high blood pressure, glucose intolerance, and myopathy) in our case have appeared after the debut of psychiatric symptoms [6]. It is worth to mention again that regular and complete physical examination is crucial when assessing patients with first-time or unexpected psychiatric symptoms.

In this case, psychiatric manifestations were signalling the onset of the disease, and although initially no organic illness was suspected as the cause of the patient's symptomatology, the subsequent clinical evolution as well as the continuation of the study revealed an endocrinological origin. In Cushing's syndrome with psychiatric symptoms, psychopharmacological treatment is considered to be of support to alleviate the symptoms that may appear both as a consequence of the disease and as adaptive symptoms.
